# Global research hotspots and trends in anti-inflammatory studies in dry eye: a bibliometric analysis (2004–2024)

**DOI:** 10.3389/fmed.2024.1451990

**Published:** 2024-11-28

**Authors:** Shan Wang, Wei Zheng, Ting Li, Dongxu Yu, Qi Zhang, Yuan Ju, Lijuan Wei

**Affiliations:** ^1^College of Chinese Medicine, Changchun University of Chinese Medicine, Changchun, Jilin, China; ^2^Ophthalmology Department, Affiliated Hospital of Changchun University of Traditional Chinese Medicine, Changchun, Jilin, China

**Keywords:** dry eye, anti-inflammatory, bibliometric, CiteSpace, R-bibliometrix, VOSviewer

## Abstract

**Background:**

Inflammation plays a crucial role in the pathophysiology of dry eye (DE). This study aims to provide a comprehensive overview of the current status, hotspots and trends in DE anti-inflammatory research through bibliometric analysis.

**Method:**

All publications were searched using the Web of Science Core Collection (WoSCC) database from 2004 to 2024. Bibliometric analyses were performed using VOSviewer, R-bibliometrix, and CiteSpace, and data were managed using Microsoft Office Excel 2019.

**Results:**

There were 603 papers published between 2004 and 2024 included in this study, with the number of papers increasing each year. The United States was the major contributor, with the largest number of publications and the greatest impact. Baylor College of Medicine was the most influential research institution. Pflugfelder, Stephen C. and Tsubota, Kazuo were the most prolific authors in this area of research, while Dana, Reza was the most cited author in the field with the highest impact. The Journal with the highest number of publications was *Investigative Ophthalmology & Visual Science*, while the highest impact journal was *Ocular Surface*. Research hotspots were focused on the mechanisms of inflammation in DE and interventions for anti-inflammatory therapy. Future studies would favor more inflammation-related targeted therapies and physical therapies.

**Conclusion:**

This study is the first bibliometric analysis to comprehensively summarize research trends and developments in DE anti-inflammatory treatments, pointing out recent research frontiers and hot directions for scholars studying DE anti-inflammatory treatments.

## 1 Introduction

DE is a common disease in ophthalmology, with the exception of refractive error and cataract, with a global prevalence of 5–50% and a higher prevalence in women than in men (1). As a chronic disease, the management of DE imposes a significant economic burden on society, with annual healthcare expenditures of approximately $3.84 billion in the United States (2), and more than $10.4 billion per year in China (3). DE is a multifactorial disease whose core mechanisms are tear film instability, ocular surface hyperosmolarity, ocular surface inflammation, and neurosensory abnormalities, among others, in which chronic activation of inflammation can lead to a vicious cycle of DE inflammation, which is considered to be the key pathogenesis of DE (4). Immune mechanisms play a key role in regulating the ocular surface environment in DE (5). The increased expression of several inflammatory factors such as interleukin (IL)-1 beta, IL-6, IL-10, interferon (IFN)-gamma, tumor necrosis factor (TNF)-alpha, has been detected in the tear fluid of patients with DE, and was positively correlated with disease severity (6, 7). Inflammation-related signaling pathways have been found to be closely related to the pathogenesis of DE (8). Activation of the NLRP 3 inflammasome plays a vital role in the immune response to DE. NLRP3 inflammatory and its downstream inflammatory factors caspase-1, IL-1 β, and IL-18 are detected as an elevated expression in the tear fluid of patients with DE (9). Targeting NLRP 3/Caspase-1/GSDMD can inhibit the pyrogenesis of corneal epithelial cells, attenuate tissue inflammation, and reduce the loss of conjunctival cup cells (10). Located upstream of inflammation, ROS can initiate the expression of NLRP3, which plays a central role in the vicious cycle of DE inflammation (11). In addition, activation of the JAK-STAT signaling pathway (12) and a reduction in the number of conjunctival cup cells were detected in an animal model of DE; and TLR agonists (13) resulted in corneal epithelial loss and thinning. The cGAS-STING signaling pathway is a new innate immune pathway that has been identified in recent years in ocular surface epithelial cells. Under environmental stress, the activation of the cGAS-STING pathway can exacerbate downstream inflammatory responses and ocular surface damage (14). All of these inflammation-related pathways were involved in the formation of DE.

Nowadays, anti-inflammatory treatments such as corticosteroids, nonsteroids, and immunosuppressants have been included in several DE treatment guidelines (15, 16). However, these drugs have some side effects and low bioavailability. Therefore, many studies have focused on the mechanisms of inflammation in DE and explored new anti-inflammatory therapies, such as dual-atom nanozyme eye drops (17), targeting the ROS-NLRP3-IL-1β Signaling Axis (18), and targeting the inflammatory pathway the NF-κB Pathway (19) to inhibit ocular surface inflammation and restore homeostasis.

Bibliometrics is a method of literature analysis that allows for a comprehensive analysis and visualization of authors, keywords, journals, countries, institutions, references, etc. in a field of study, thus helping us to quickly understand the development trends (20) and research hotspots in the field (21). Bibliometrics are now widely used in medical fields such as endocrine and metabolic diseases (22), immune disorders (23), oncological diseases (24), orthopedic diseases (25), etc., and show great potential (21).

Anti-inflammatory therapy is currently a hot topic in DE treatment; however, there are currently no bibliometric analyses in this area. To fill this knowledge gap, this study applied CiteSpace (20), VoSviewer (26), and the R package “bibliometrix” (27) to conduct a bibliometric analysis of publications in DE anti-inflammatory studies over the past two decades (2004–2024) to identify key contributors and the current state of research and to foresee research trends and prospects in the field. Preferred Reporting Items for Bibliometric Analysis (PRIBA) is the first PRISMA Checklist of bibliometric studies (28). The PRIBA was referenced in our study, and we hope to provide some reference and insights for scholars studying this area.

## 2 Methods

### 2.1 Search strategy

**Figure 1:**
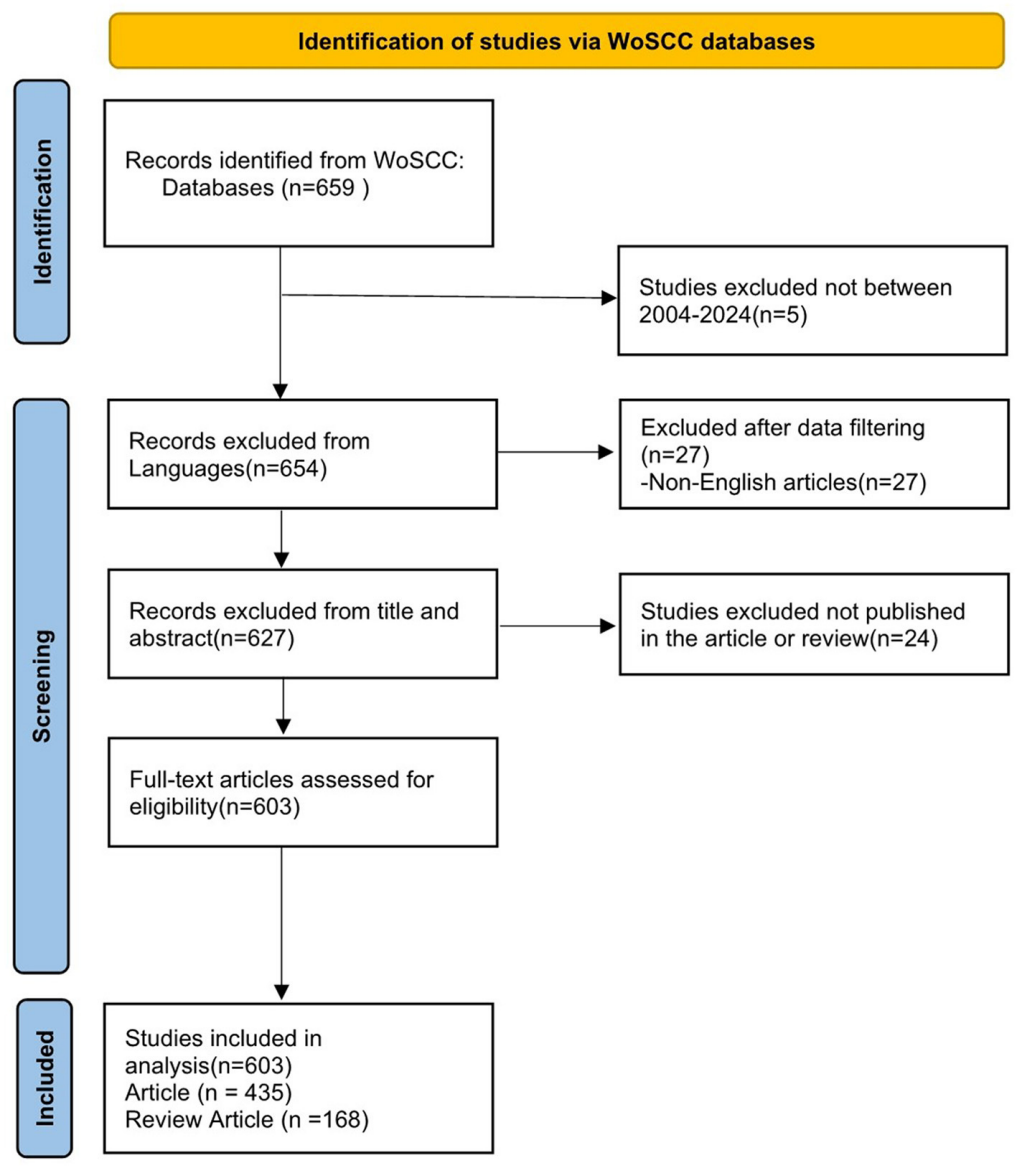
Publications screening flowchart.

We conducted a literature search on May 5, 2024, on the WoSCC database. WoSCC is known for its high quality scientific journals and comprehensive citation content. It is also the most widely used database in bibliometric studies currently (29–31). The search formula was (TS=(Dry Eye^*^ OR Dry Eye Syndrome^*^ OR Dry Eye Disease^*^ OR Evaporative Dry Eye^*^)) AND (TS=(anti inflammatory^*^ OR anti-inflammatory^*^ OR anti-inflammation^*^)) AND DOP=(2004-01-01/2024-05-05). The file types are set to “Article” and “Reviews”. The language of the article is limited to English. Literature searching was carried out independently by two authors (S W and DX Y), and the third author (T L) decided in case of disagreement. Finally, the retrieved documents are extracted into a text file with “full record and cited references”, and the screening process is shown in Figure 1. All search strategies and screening records can be found in the Appendix.

### 2.2 Data analysis

A total of 603 papers were screened and included in our study. We used Microsoft Office Excel 2019 to manage and analyze the statistics of the annual volume of publications, country regions, institutions, authors, keywords and other data, and conducted the linear regression analysis of the volume of publications and other data. VOSviewer (1.6.18), CiteSpace (version 6.2.R3), and the R package “bibliometrix” were used for bibliometric analysis and visualization of the screened results.

VOSviewer, CiteSpace, and R-bibliometrix are all popular bibliometric analysis tools. VOSviewer visualizes and analyzes the paper, presenting the various parts of the research area in different plots, such as annotation view, density view, clustered density view, and scatter view (26). VOSviewer was applied to produce a visual mapping to visualize and analyze the collaboration network for country regions, research institutions, authors, journals, and keywords to understand the mutual collaborative relationships. The connecting line between each other meant that there was cooperation between them. Circles represented the target of the study, and their size indicated the frequency of occurrence. The lighter the color, the closer to the present year in which the target of the study appeared; conversely, the darker the color, the further away from the present. We set different parameters according to different research objectives to better visualize and present the results. Specific parameter settings were shown in the legend. In addition, we performed cluster analysis on the visual maps of literature and keywords by combining with Pajek software, where different colors represent different clusters, and the same clusters are arranged in vertical rows, in order to facilitate our generalization and summary of them.

CiteSpace's burst detection algorithm identifies emerging research frontiers (20), enabling researchers to have a deeper understanding of the hotspots and directions of research in the field. CiteSpace was applied to analyze the bursts of keywords. The red color represented the time of occurrence and the length indicated the year of duration. With the application of keyword burst analysis, we could find out when the topics represented by the keywords appear and be active in order to predict the hotspots of the research.

R-bibliometrix enables comprehensive scientific mapping analysis of the documents (27). R-bibliometrix was used to analyze national collaborations, annual publication trends in the Top 5 journals, Global Citation Score (GCS) and Local Citation Score (LCS) analyses, and trending theme analyses. The impact of the literature can be evaluated by the two scores GCS and LCS. By analyzing the frequency of keyword appearances and duration, trending theme analysis can predict the development trend of the theme.

## 3 Results

### 3.1 Quantitative analysis of publication

**Figure 2:**
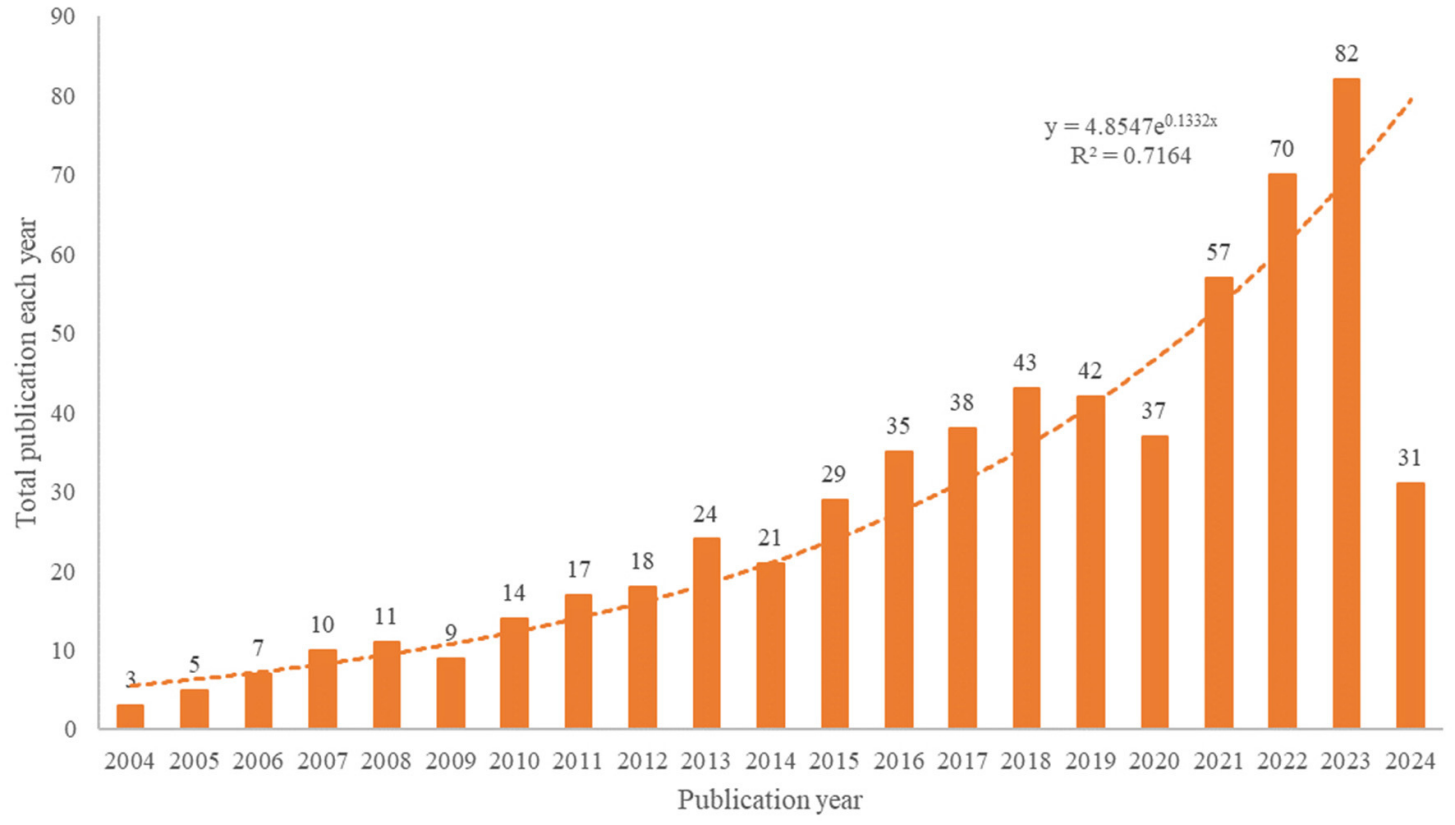
Annual output of research of anti-inflammatory studies in DE from 2004 to 2024.

The number of publications and their trends reflect the state of research and development in the field of study. According to our search strategy, a total of 603 studies on anti-inflammatory studies for DE were conducted from 2004 to 2024, and the annual number of publications and growth rates are shown in Figure 2.

In terms of the growth rate of the number of publications per year, the entire period can be divided into two parts: phase 1 (2004–2013) and phase 2 (2014–2023). The first phase has a relatively small number of publications and is the initial phase of research on anti-inflammatory treatments for DE. There has been a growing body of anti-inflammatory research in DE since 2014 when Wei et al. (32) suggested that the core mechanism of DE is inflammation, and this research has entered its second phase. Rapid growth in 2020–2023 shows that the sector has received increasing attention in recent years. The decrease in the number of publications in 2024 may be due to the fact that our search ended on May 5, 2024. The trend of annual publications was analyzed by applying Microsoft Excel 2019, and the fitted equation was y = 4.8547e^0.1332x^, R^2^ = 0.7164, which has a well-fitted nature and conforms to the Price curve. Overall, the number of annual publications in the field of DE anti-inflammatory has steadily increased.

### 3.2 Distribution and co-authorship of countries/regions

**Figure 3:**
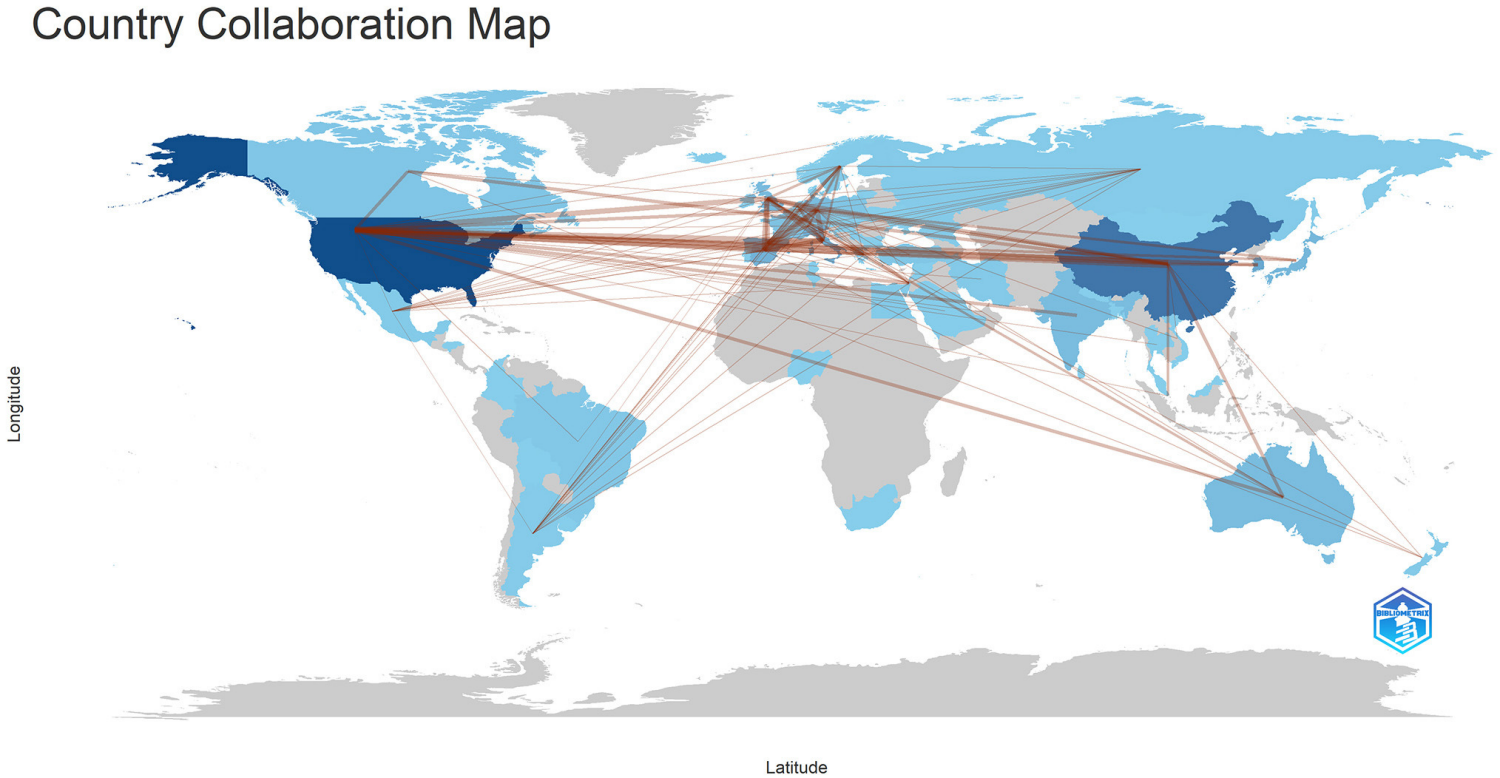
The geographical distribution of collaborations among different countries/regions. Min edges = 2.

**Figure 4:**
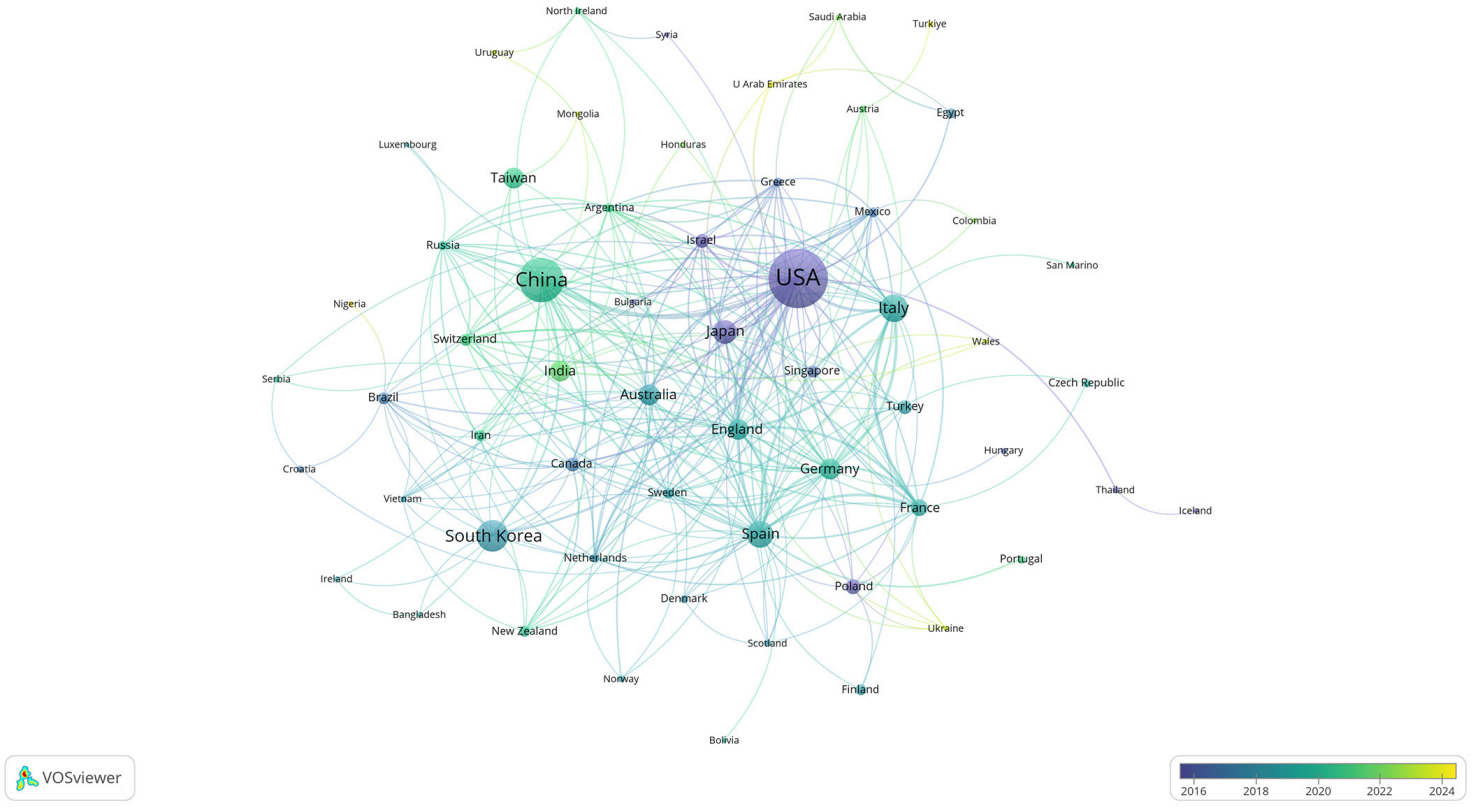
Co-authorship network of countries/regions in anti-inflammatory studies in DE (the minimum number of documents of a country/region was set as 1; 68 of the 68 countries involved in anti-inflammatory studies in DE).

Publications came from 68 countries, with the United States having the largest publications and influence (Table 1). The distribution of the map of national and regional collaboration networks was shown in Figure 3. Subsequently, we constructed co-authorship networks based on the number of publications in each country, the time of publication and the collaboration between them (Figure 4). Each node's size represents the number of articles published in the respective country/region, and the connecting lines between the nodes indicate the existence of collaboration between the two, with the thickness of the lines characterizing the strength of the collaboration. There is a lot of extensive cooperation between different countries. We noted that countries that work closely with others generally have higher levels of influence and that there may be a potential link between them. For example, The United States had the most intense cooperation with China, as well as with several European countries, such as Italy and the United Kingdom (Table 2). We noted that German publications were at the forefront of influence, although not large in number. This may be related to the close cooperation between Germany and other countries in the top 10 in terms of influence, such as Spain, Italy and the United Kingdom.

**Table 1:** Top 10 countries/regions contributing to volume and influence of publications in anti-inflammatory studies in DE.

**Rank**	**Country**	**Documents**	**Rank**	**Country**	**Citations**
1	USA	201	1	Usa	9,329
2	China	112	2	China	2,500
3	South Korea	56	3	Germany	2,217
4	Italy	45	4	Spain	1,981
5	Spain	40	5	Italy	1,812
6	Japan	33	6	England	1,655
7	Australia	26	7	South Korea	1,450
8	England	26	8	Australia	1,374
9	Germany	26	9	Canada	1,325
10	India	26	10	Japan	948

**Table 2:** Top 10 most frequent cooperation countries in anti-inflammatory studies in DE.

**From**	**To**	**Frequency**
China	USA	18
Italy	France	9
Italy	Germany	8
Spain	Germany	8
Spain	United Kingdom	8
United Kingdom	Germany	8
Usa	Italy	8
Usa	United Kingdom	8
Italy	United Kingdom	7
Spain	France	7

### 3.3 Distribution and co-authorship of research organizations

**Figure 5:**
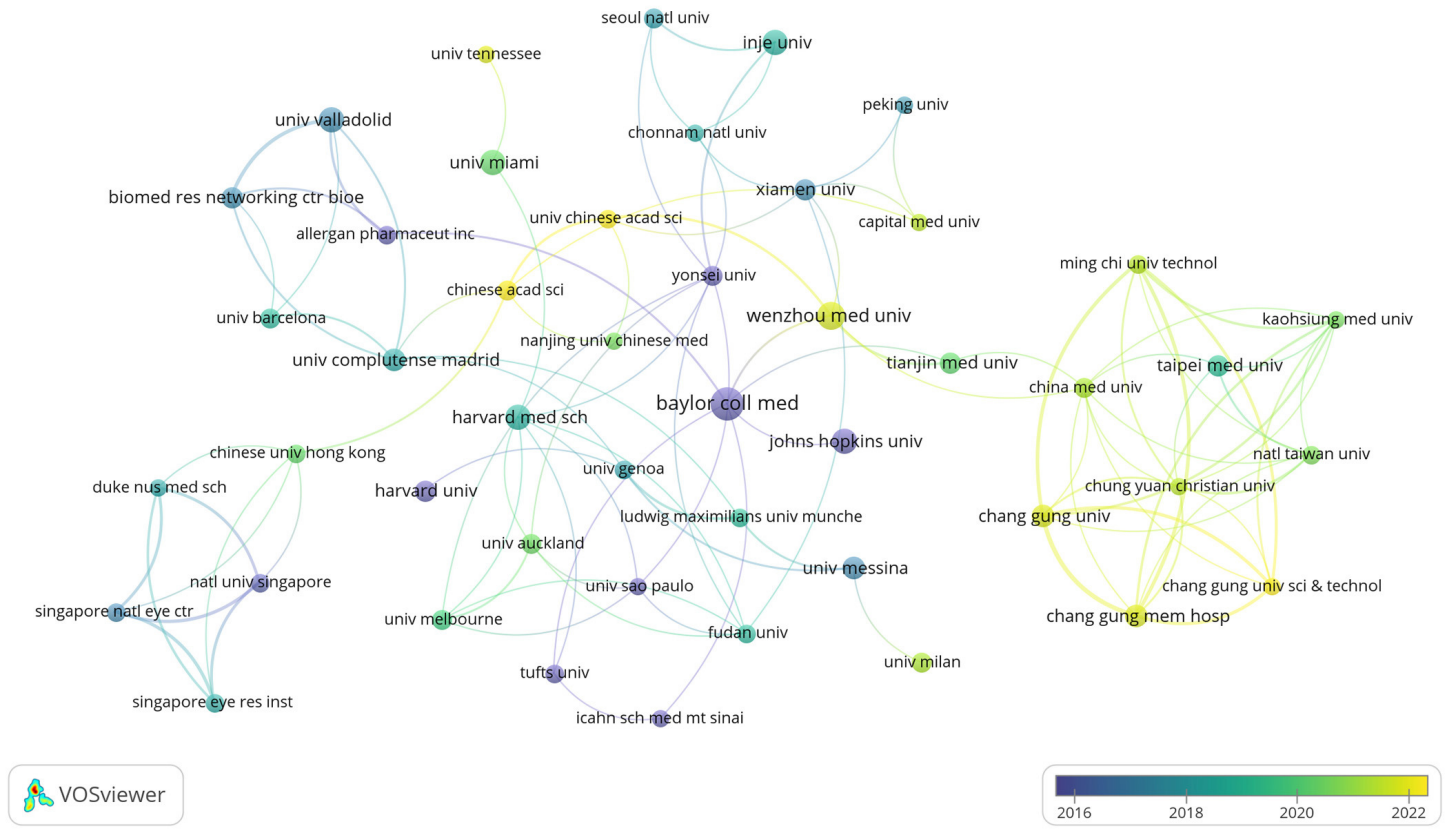
Co-authorship network of research organizations in anti-inflammatory studies in DE (the minimum number of documents of a research organization was set as 5; 54 of the 957 research organizations involved in anti-inflammatory studies in DE).

The publications retrieved in this study came from 957 research institutions. The top 10 institutions in publications contributed 119 articles (Table 3). Regarding the number of publications, Baylor College of Medicine published the highest number of papers, followed by Keio University and Wenzhou Medical University. In terms of publication impact, Baylor College of Medicine is in first place, with Harvard Medical School and Universidade de São Paulo following. Co-authorship networks were constructed for the number of publications and relationships of the research organizations in Figure 5. Each node's size represents the number of articles published by the respective research organization, and the connecting lines between the nodes represent the existence of collaboration among them. Results of the analysis of the institutional co-authorship network showed close cooperation between institutions such as Baylor College of Medicine, Harvard Medical School, and Universidade de São Paulo.

**Table 3:** Top 10 research organizations contributing to volume and influence of publications in anti-inflammatory studies in DE.

**Rank**	**Organization**	**Documents**	**Rank**	**Organization**	**Citations**
1	Baylor Coll Med	19	1	Baylor Coll Med	1,260
2	Keio Univ	14	2	Harvard Med Sch	1,032
3	Wenzhou Med Univ	13	3	Univ Sao Paulo	895
4	Harvard Med Sch	11	4	Yonsei Univ	867
5	Inje Univ	11	5	Univ Auckland	815
6	Johns Hopkins Univ	11	6	Univ Melbourne	811
7	Univ Miami	11	7	Fudan Univ	809
8	Univ Valladolid	11	8	Allergan Pharmaceut Inc	555
9	Chang Gung Mem Hosp	9	9	Harvard Univ	552
10	Chang Gung Univ	9	10	Tufts Univ	501

### 3.4 Distribution and co-authorship of authors

**Figure 6:**
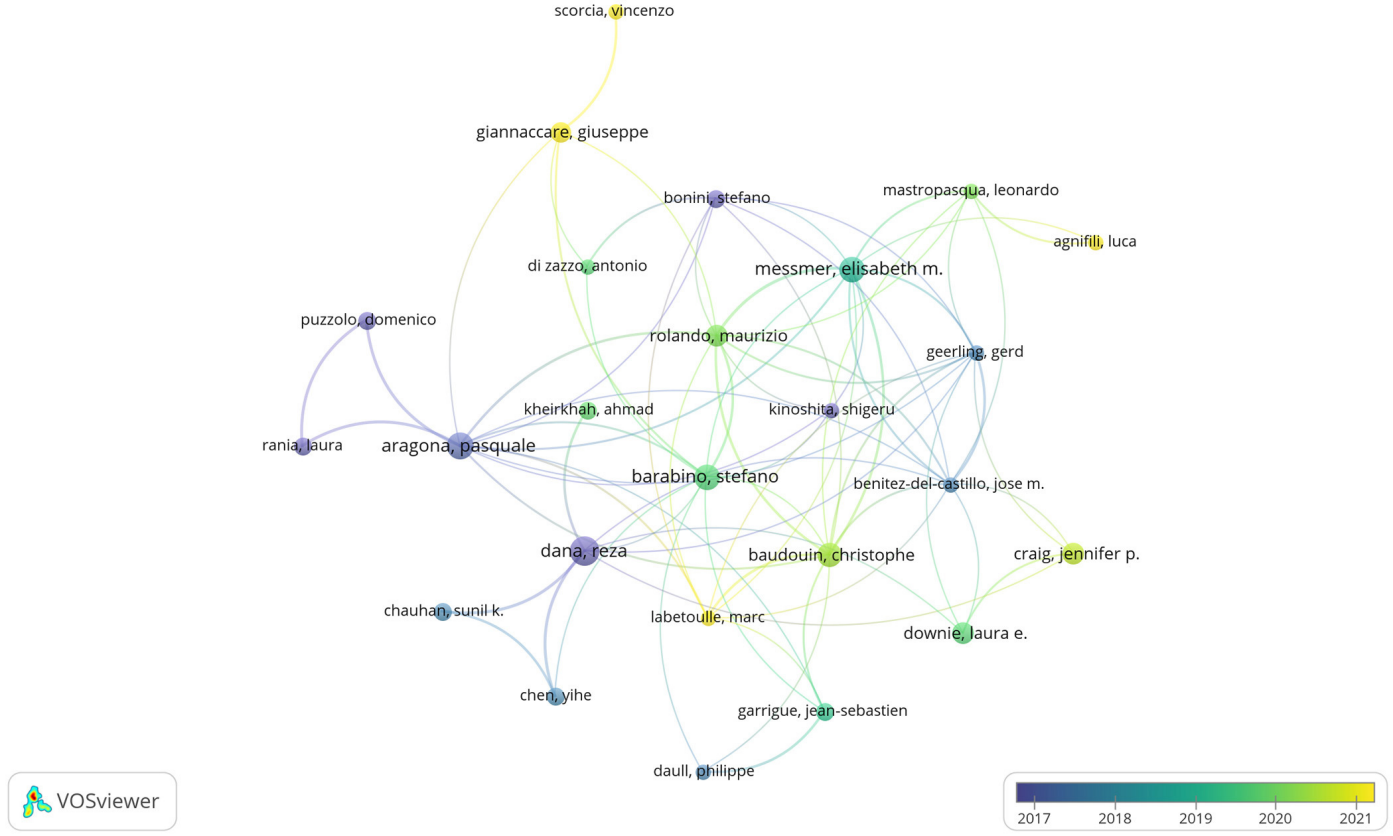
Co-authorship network of authors in anti-inflammatory studies in DE (the minimum number of documents of an author was set as 3; 105 of the 3,005 authors involved in anti-inflammatory studies in DE).

There were 3,005 authors involved in the study of anti-inflammatory treatments for DE. The top 10 most productive and influential authors in the field were listed in Table 4. The total number of publications by the top ten authors was 96, accounting for 15.92% of the total number of publications in the field. The largest number of postings were made by Pflugfelder, Stephen C., and Tsubota, Kazuo, both with 13 articles. In terms of influence, Dana, Reza had the highest influence, followed by Benitez-Del-Castillo, Jose M. and Geerling, Gerd. The co-authorship network of authors of anti-inflammatory studies in DE is shown in Figure 6. While the size of each node represents the number of articles published by the author, the connecting lines between the nodes represent the existence of collaboration among them. We noted that Dana Reza maintains a close collaboration among other authors and had the most impact despite not having the most publications. We also noted that Benitez-Del-Castillo Jose M. and Geerling Gerd had emphasized collaborations with other authors in recent years and were ahead of the influence, even if they were not in the top ten in terms of publications.

**Table 4:** Top 10 authors contributing to volume and influence of publications in anti-inflammatory studies in dry eye.

**Rank**	**Author**	**Documents**	**Rank**	**Author**	**Citations**
1	Pflugfelder Stephen C.	13	1	Dana Reza	1,246
2	Tsubota Kazuo	13	2	Benitez-Del-Castillo Jose M.	1,043
3	De Paiva Cintia S.	11	3	Geerling Gerd	1,043
4	Dana Reza	10	4	De Paiva Cintia S.	833
5	Aragona Pasquale	9	5	Messmer Elisabeth M.	826
6	Li De-Quan	9	6	Pflugfelder Stephen C.	811
7	Barabino Stefano	8	7	Downie Laura E.	805
8	Messmer Elisabeth M.	8	8	Craig Jennifer P.	804
9	Tseng Ching-Li	8	9	Li De-Quan	760
10	Asbell Penny A.	7	10	Stern Michael E.	625

### 3.5 Journal analyses

**Figure 7:**
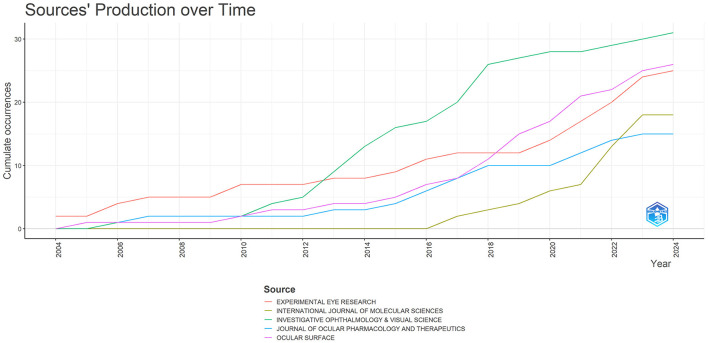
Trends in annual publication volume for the top five journals by publication volume in anti-inflammatory studies in DE.

**Figure 8:**
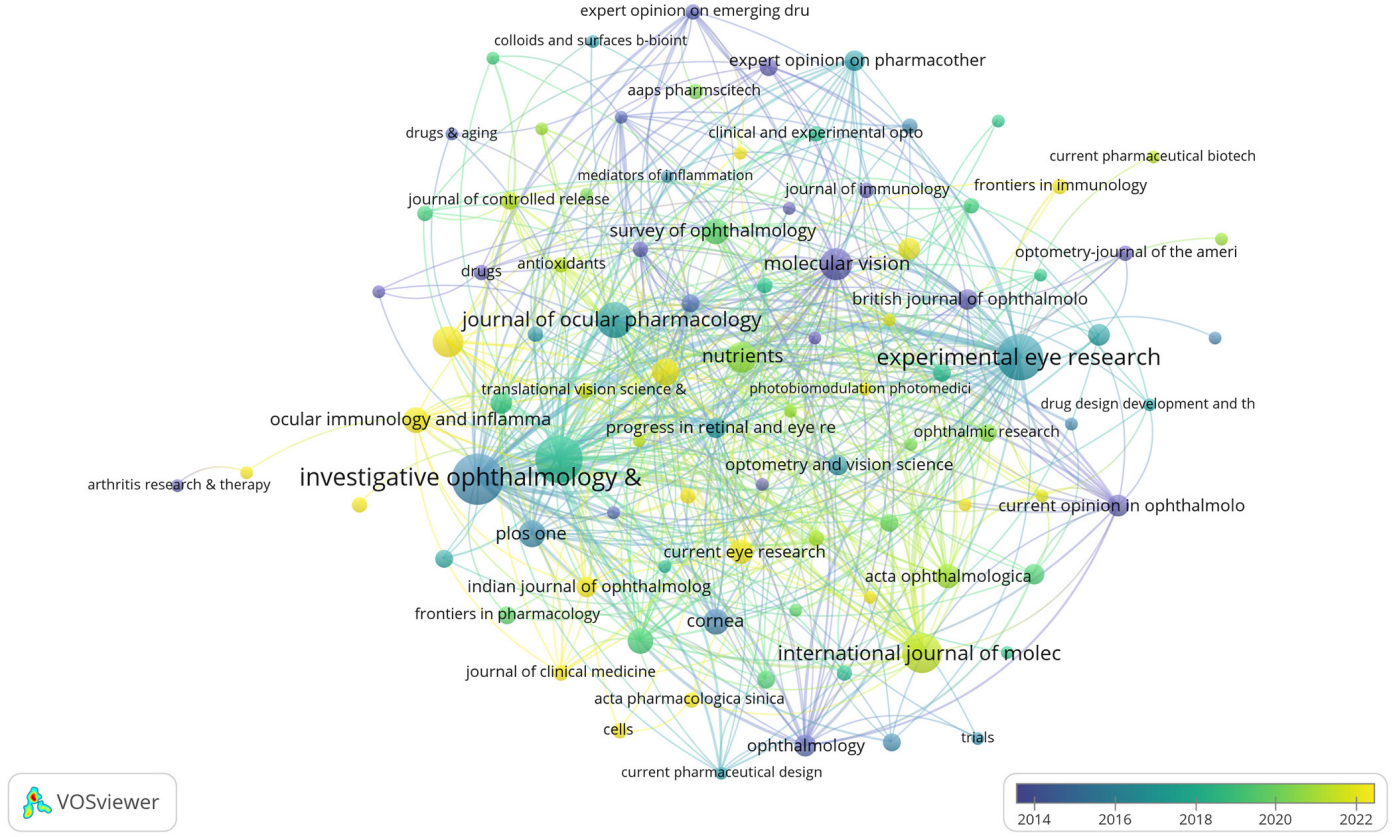
Citation network of journals in anti-inflammatory studies in DE (the minimum number of journal documents was set as 2; 97 of the 245 journals involved in anti-inflammatory studies in DE).

The DE anti-inflammatory studies retrieved in this study were published in 245 journals. The top 10 most prolific and influential journals in the field were presented in Table 5. The top five journals in terms of productivity were “*Investigative Ophthalmology & Visual Science*,” “*Ocular Surface*,” “*Experimental Eye Research*,”, “*International Journal Of Molecular Sciences*,” and “*Journal Of Ocular Pharmacology And Therapeutics*,” with 31, 26, 25, 18, and 15 articles, respectively. The annual publication volume of the top five journals is shown in Figure 7, and all of these journals have shown an increasing trend in annual publication volume in recent years. “*Ocular Surface*” stood out as the most influential journal in the field with 1,529 citations. The citation network of journals is illustrated in Figure 8. Whereas, the size of each node corresponds to the number of articles published in the research journals, the connecting lines between the nodes represent the existence of a cooperative relationship between the two, with the strength of the connecting lines reflecting the degree of cooperation.

**Table 5:** Top 10 journals contributing to volume and influence of publications in anti-inflammatory studies in DE.

**Rank**	**Journals**	**Documents**	**JCR (2023)**	**JIF RANK**	**IF**	**Rank**	**Journals**	**Citations**	**JCR (2023)**	**JIF RANK**	**IF**
1	Investigative Ophthalmology & Visual Science	31	Q1	6/95	5	1	Ocular Surface	1,529	Q1	4/95	5.9
2	Ocular Surface	26	Q1	4/95	5.9	2	Investigative Ophthalmology & Visual Science	1,162	Q1	6/95	5
3	Experimental Eye Research	25	Q1	18/95	3	3	Experimental Eye Research	991	Q1	18/95	3
4	International Journal of Molecular Sciences	18	Q1	66/313	4.9	4	Biomedicine & Pharmacotherapy	788	Q1	21/189	6.9
5	Journal of Ocular Pharmacology and Therapeutics	15	Q2	44/95	1.9	5	Progress In Retinal And Eye Research	614	Q1	1/95	18.6
6	Molecular Vision	12	Q4	259/313	1.8	6	Survey Of Ophthalmology	528	Q1	5/95	5.1
7	Nutrients	11	Q1	18/114	4.8	7	Ophthalmology	528	Q1	2/95	13.1
8	Pharmaceutics	11	Q1	45/354	4.9	8	Molecular Vision	513	Q4	259/313	1.8
9	Frontiers in Medicine	9	Q1	56/325	3.1	9	Current Opinion In Ophthalmology	277	Q1	18/95	3
10	PLoS ONE	9	Q1	26/73	2.9	10	International Journal Of Molecular Sciences	266	Q1	66/313	4.9

The JCR rankings can reflect the influence of the journal in its field. We reviewed the 2023 JCR rank, JIF quartile, and Journal Impact Factor (IF) of the ten journals with the highest impact in this study (Table 5). We found that 80% of the top ten journals in terms of publications belong to the Q1 region; this ratio is as high as 90% for the top ten journals in terms of impact, which reflected that both the top ten journals in terms of publications and impact are of very high quality. We hypothesized that there would be a linear correlation between the JCR of the top ten journals in this study and the impact of the articles, and the CORREL function in Excel was applied to analyze the correlation between citations and JCR rankings. But, unfortunately, the result was not exactly what we expected, the correlation coefficient of the two was −0.28, showing a weak correlation between them. This may be due to the fact that we only performed correlation analyses on the top 10 journals, which was not a sufficient sample size. Or maybe the dataset we chose was specific and could only cover the content of our research area and could not give a complete overview of the quality of the entire journal. Thus, more data is needed to explore the relationship between them.

### 3.6 Co-cited references

**Figure 9:**
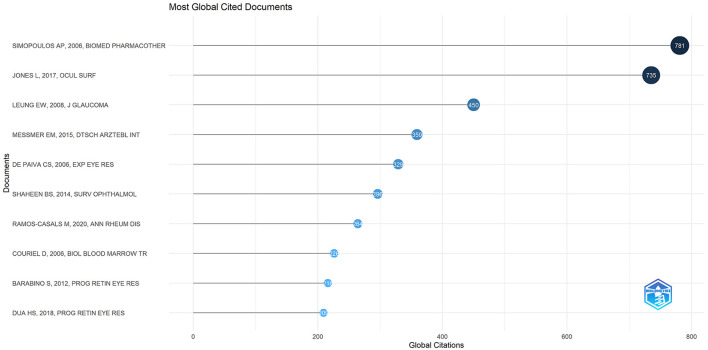
Top 10 references on GCS of anti-inflammatory studies in DE.

**Figure 10:**
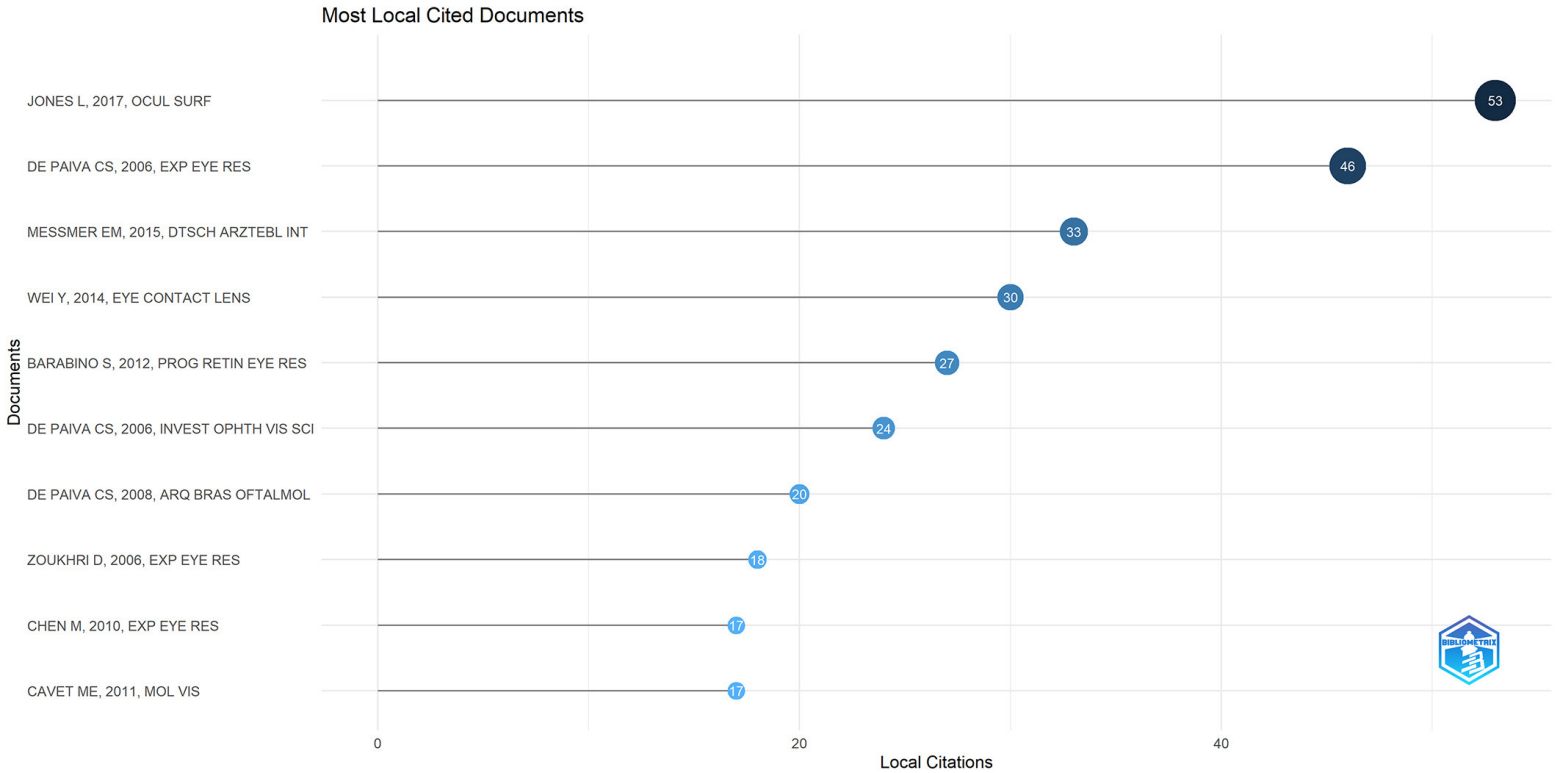
Top 10 references on LCS of anti-inflammatory studies in DE.

**Figure 11:**
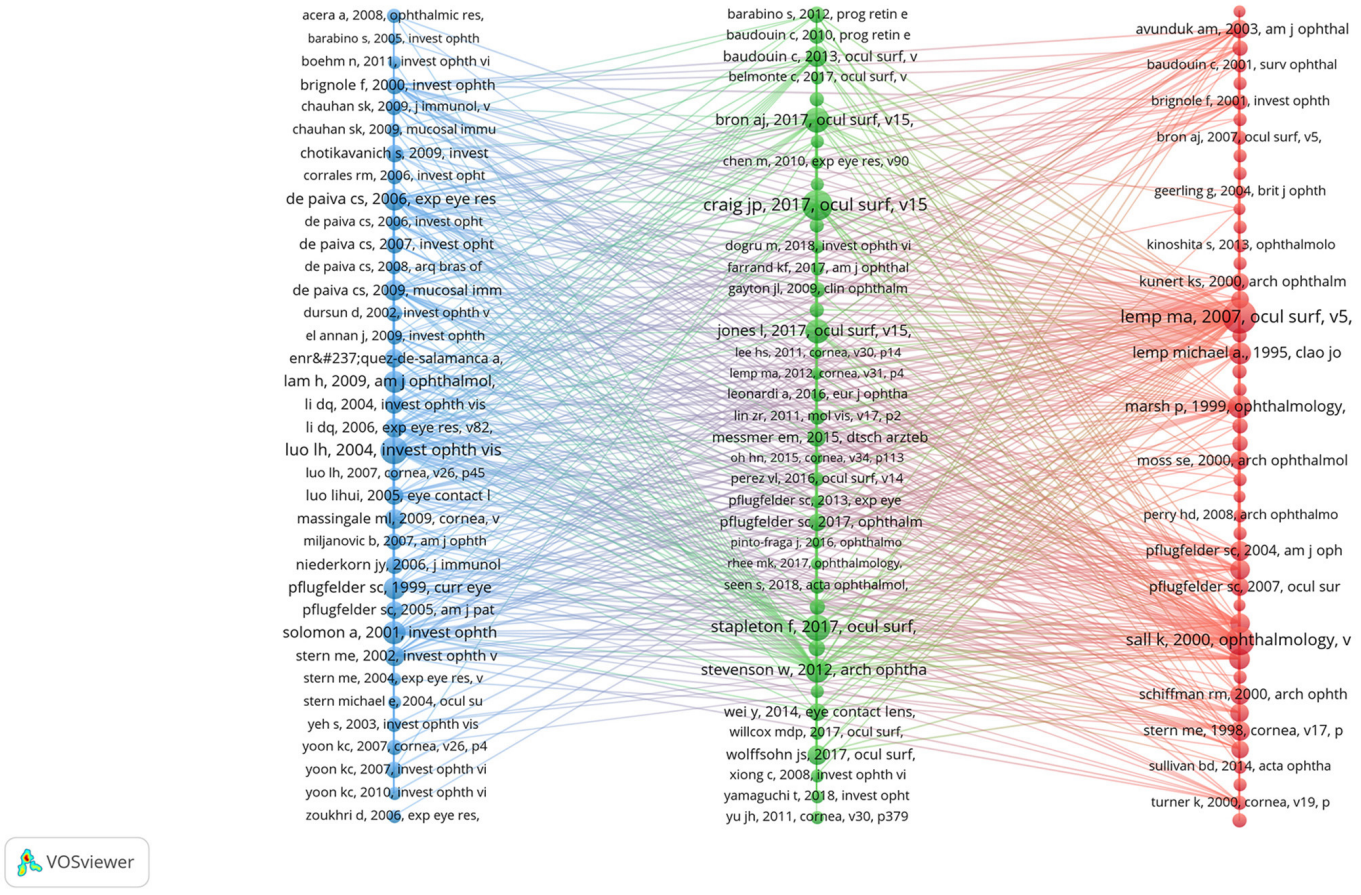
Cluster network of co-cited references produced by VOSviewer and Pajek.

There were 27,173 co-cited references on DE anti-inflammatory research in the last two decades. Cited articles can help us quickly understand the content and frontiers of our research field, and two metrics can be used to evaluate cited literature, including GCS (Figure 9) and LCS (Figure 10). The top five GCSs and LCSs were shown in Table 6, indicating that these literature are recognized by researchers in the field. We constructed a co-citation network graph of the literature, with the minimum citation frequency set at 15, and 121 documents reached the threshold. Each node represents an article and the node size represents the citation frequency. These 121 documents were clustered into three categories, represented in red, green, and blue (Figure 11). According to Figure 11, “Luo Lh, 2004, Invest Ophth” shows an active co-citation relationship with “Craig Jp, 2017, Ocul Surf” and “Lemp Ma, 2007, Ocul Surf”.

**Table 6:** The references with the top five LCS and GCS of anti-inflammatory studies in DE.

**Rank**	**Paper**	**DOI**	**Year**	**GCS**	**Rank**	**Paper**	**DOI**	**Year**	**LCS**
1	Simopoulos AP, 2006, Biomed Pharmacother	10.1016/j.biopha.2006.07.080	2006	781	1	Jones L, 2017, Ocul Surf	10.1016/j.jtos.2017.05.006	2017	53
2	Jones L, 2017, Ocul Surf	10.1016/j.jtos.2017.05.006	2017	735	2	De Paiva CS, 2006, Exp Eye Res	10.1016/j.exer.2006.02.004	2006	46
3	Leung EW, 2008, J Glaucoma	10.1097/IJG.0b013e31815c5f4f	2008	450	3	Messmer EM, 2015, Dtsch Arztebl INT	10.3238/arztebl.2015.0071	2015	33
4	Messmer EM, 2015, Dtsch Arztebl Int	10.3238/arztebl.2015.0071	2015	359	4	Wei Y, 2014, Eye Contact Lens	10.1097/ICL.0000000000000042	2014	30
5	De Paiva CS, 2006, Exp Eye Res	10.1016/j.exer.2006.02.004	2006	329	5	Barabino S, 2012, Prog Retin Eye Res	10.1016/j.preteyeres.2012.02.003	2012	27

### 3.7 Hotspots and frontiers

**Figure 12:**
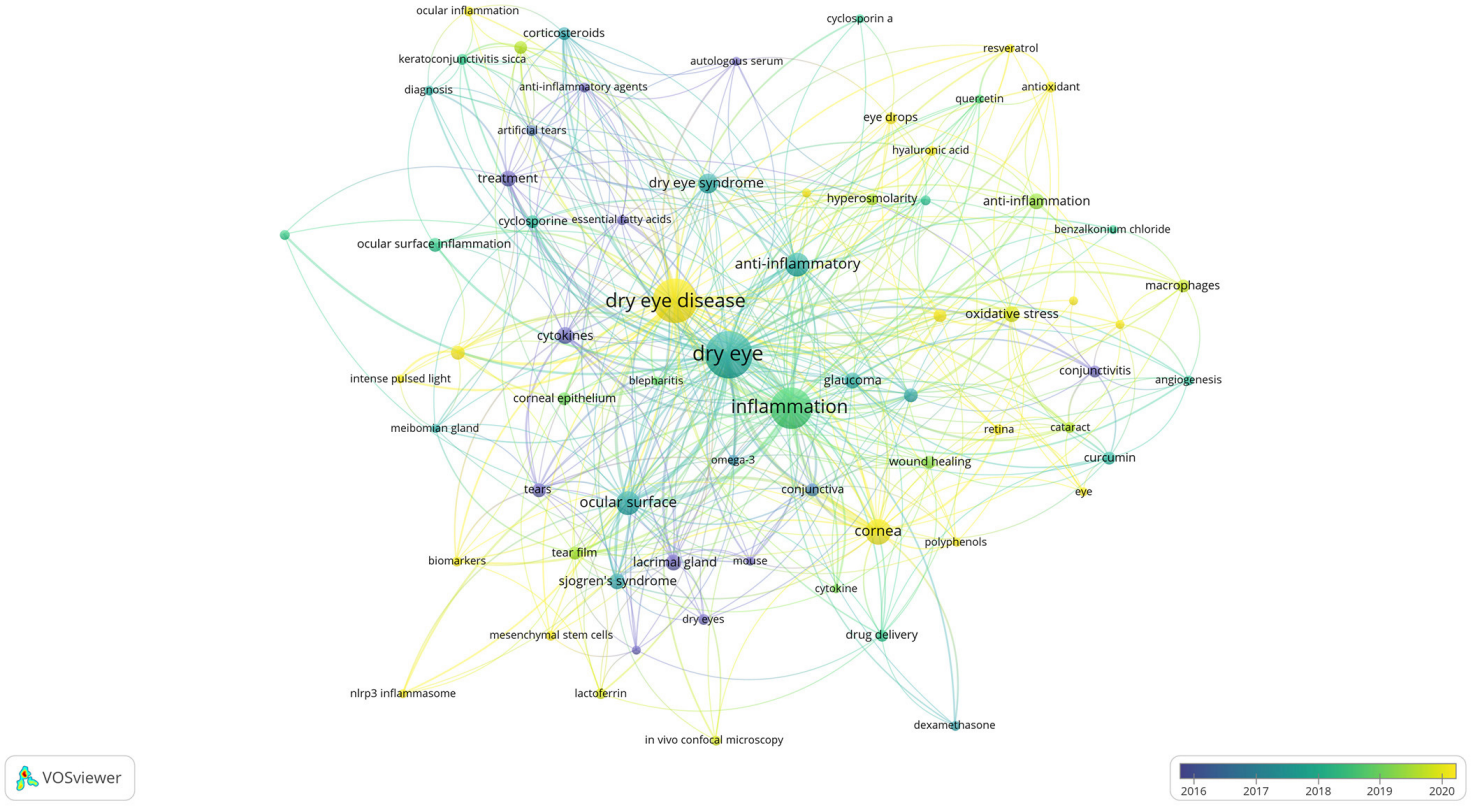
Co-authorship network of keywords in anti-inflammatory studies in DE (the minimum number of documents of a keyword was set as 5; 69 of the 1,504 keywords involved in anti-inflammatory studies in DE).

Keyword co-occurrence analysis can help us quickly understand the research hotspots in a certain field. We displayed the keywords as an overlay, with different colors representing the year of publication, and lighter colors representing the closer the publication date as shown in Figure 12, there were many emerging areas of DE anti-inflammatory research in recent years, such as “intensity pulsed light, meibomian gland dysfunction, nlrp3 inflammasome, oxidative stress, antioxidant, biomarkers, mesenchymal stem cells, eye drops,” etc., which to some extent can reflect the vigorous development of this research field. The top five core keywords according to VOSviewer's analysis of the Co-authorship network of keywords are: dry eye, inflammation, dry eye disease, ocular surface, cornea.

**Figure 13:**
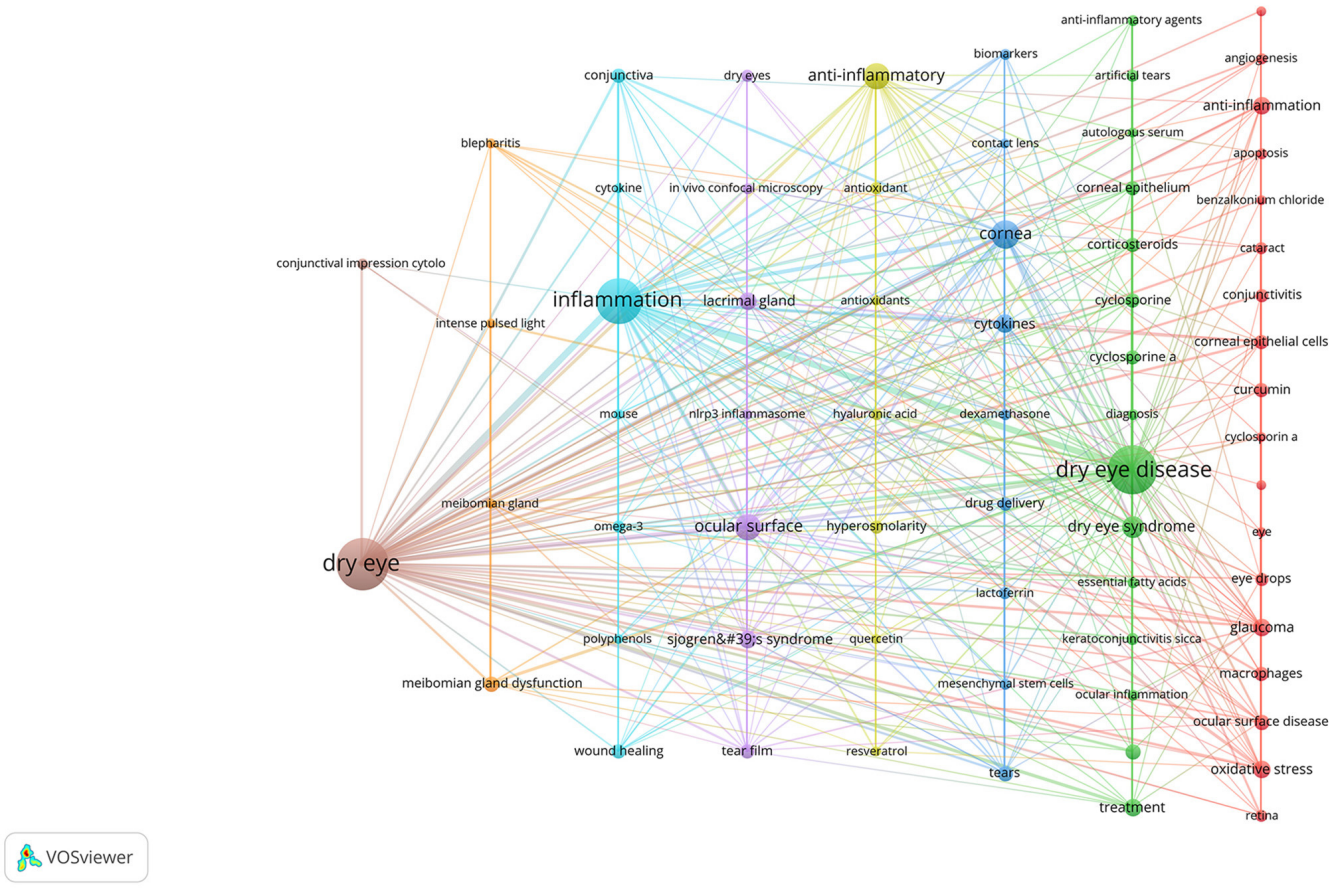
Cluster network of keywords produced by VOSviewer and Pajek. The keywords were divided into eight groups, each displayed in a different color.

We then performed keyword cluster network analysis using VOSviewer and Pajek (Figure 13), selecting 69 keywords with a frequency ≥ 5 for visualization. These keywords were clustered into eight main categories, indicated by different colors, representing eight research themes. The first group (red) included: glaucoma, oxidative stress, ocular surface disease, curcumin, corneal epithelial cells, macrophages, conjunctivitis, eye drops, cataract, retina, angiogenesis, apoptosis, age-related macular degeneration (AMD), benzalkonium chloride, cyclosporin a. The second group (green) included: ocular surface inflammation, corticosteroids, cyclosporine, corneal epithelium, cyclosporine a, artificial tears, keratoconjunctivitis sicca, anti-inflammatory agents, diagnosis, essential fatty acids, ocular inflammation, autologous serum. The third group (dark blue) included: cornea, cytokines, tears, drug delivery, lactoferrin, biomarkers, dexamethasone, mesenchymal stem cells, contact lens. The fourth group (yellow) included: hyperosmolarity, antioxidant, hyaluronic acid, antioxidants, quercetin, resveratrol. The fifth group (purple) included: ocular surface, Sjogren's syndrome, lacrimal gland, tear film, dry eyes, *in vivo* confocal microscopy, nlrp3 inflammasome. The sixth group (light blue) included: conjunctiva, wound healing, omega-3, cytokine, mouse, polyphenols. The seventh group (orange) included: meibomian gland dysfunction, blepharitis, intense pulsed light, meibomian gland. The eighth group (brown) included: conjunctival impression cytology.

**Figure 14:**
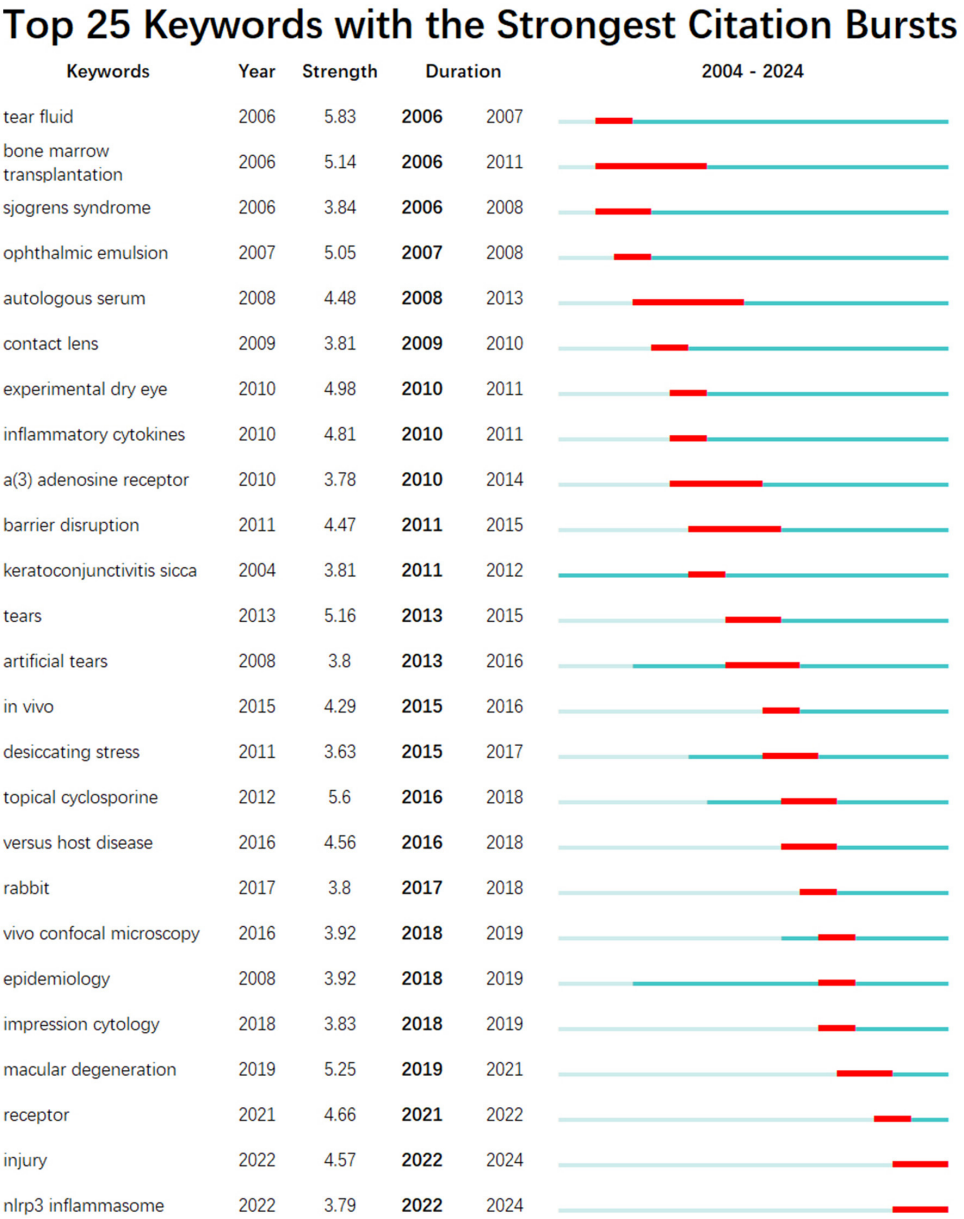
The top 25 keywords with the strongest citation bursts in anti-inflammatory studies in DE. The red bold line represents the burst years.

The top 20 keywords with a frequency of more than 10 were shown in Table 7. To further understand the hot trends and progress in this research field, we conducted a burst analysis of keywords in this field through CiteSpace. Different colors represent the status of the keywords at the corresponding time, red means that the keyword has appeared. The top 25 keywords with the strongest burst of citations in the field of DE anti-inflammatory treatments between 2004 and 2024 were shown in Figure 14, along with the intensity and timing of their emergence. As can be seen from the figure, “expression cytology,” “macular degeneration,” “receptor,” “injury,” and “nlrp3 inflammasome” were emerging and active themes in recent years, and probably represent the current hotspots of anti-inflammatory research in DE.

**Table 7:** High occurrences keywords in anti-inflammatory studies in DE.

**Rank**	**Keyword**	**Occurrences**	**Rank**	**Keyword**	**Occurrences**
1	Dry eye	123	11	Anti-inflammation	14
2	Dry eye disease	112	12	Lacrimal gland	14
3	Inflammation	96	13	Oxidative stress	14
4	Cornea	36	14	Treatment	14
5	Ocular surface	33	15	Meibomian gland dysfunction	11
6	Anti-inflammatory	31	16	Ocular surface disease	11
7	Dry eye syndrome	23	17	Ocular surface inflammation	11
8	Cytokines	16	18	Tears	11
9	Glaucoma	15	19	Corticosteroids	10
10	Sjogren's syndrome	15	20	Curcumin	10

**Figure 15:**
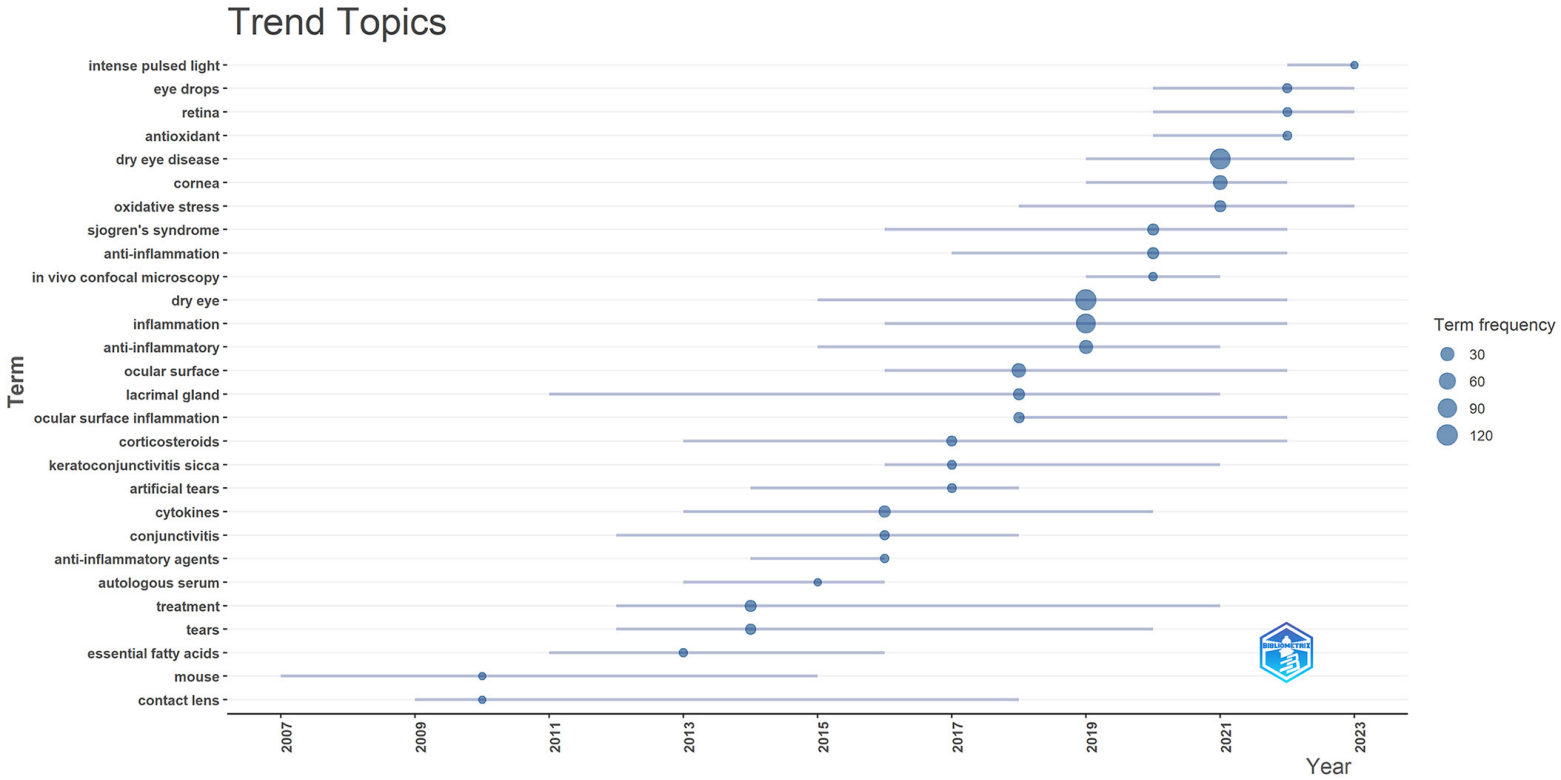
Trend topics in anti-inflammatory studies in DE. The *X*-axis represents the year and the *Y*-axis presents the keywords, with the size of the circle on each keyword representing its frequency of occurrence.

We applied “Biblioshiny” to trend topics analysis of author keywords. The horizontal axis of the graph represents time, each bubble represents a topic, the size of the bubble represents the frequency of occurrence, the position of the bubble is set about the median of the period in which the keyword appeared, and the bars represent the first and third quartiles of the time in which the keyword appeared. The evolution of popular research topics in DE anti-inflammatory treatment over time was shown in Figure 15, from which it can be seen that pulsed light therapy had received close attention during the last 2 years.

With the above results, we summarized the keywords into four main groups. These were the hotspots that had been closely focused on in the field for the last 20 years and had a continuing trend in the future. Group 1: Target structures for DE occurrence and treatment (including cornea, ocular surface, conjunctiva, lacrimal gland, and tears). Group 2: Ocular diseases closely related to DE (including glaucoma, dry syndrome, and blepharospasmal gland dysfunction). Group 3: Mechanism of inflammation in DE (focusing on oxidative stress, NLRP3). Group 4: Anti-inflammatory treatment aspects (focus is mostly on anti-inflammatory drugs, antioxidant drugs, drug delivery modalities, physical therapy).

## 4 Discussion

### 4.1 General information

This study was the first to use a bibliometric approach to identify the current state of research, research hotspots, and trends in the field of DE anti-inflammatory research. We performed statistical analyses and visualizations of highly productive countries, research organizations, authors, journals, references and keywords. After screening, a total of 603 papers were included in this study during 2024–2024. The number of annual publications in the field has been on an overall increasing trend over the past two decades and has grown even more rapidly in the last decade. This indicated that the field of DE anti-inflammatory research was receiving a lot of attention from scholars.

Well-organized cooperation can contribute to the development of the discipline, and this study found that there are strong links between countries, institutions, and authors, which can facilitate the sharing of research results and form a lasting development of the discipline. The results of the study showed that the countries with the highest number of DE anti-inflammatory research articles published were the USA, China, and South Korea. The top three research institutions for the number of publications are Baylor College of Medicine, Keio University, and Wenzhou Medical University. Pflugfelder, Stephen C. had the most publications in the field of DE anti-inflammatory research and was one of the top six co-cited authors, highlighting his contribution to the field of research. Pflugfelder, Stephen C. from the Ocular Surface Center, Department of Ophthalmology, for many years, focused on DE immunological, anti-inflammatory, and clinical research. He proposed anti-inflammatory treatment for DE as early as 2003 (33), coined the term “flare” in 2020 (34), and summarized and analyzed the Innate and adaptive immune responses in DE. “Flare” is caused by a complex inflammatory cascade response, and biomarkers associated with flare may be potential new therapeutic targets. Dana Reza, with 1,246 citations, is the most cited author in the field of DE anti-inflammatory research and has been focusing on the immunological aspects of DE and its targeted therapies. His team designed a photo-crosslinked adhesive patch (GelPatch) (35) for drug delivery and sustained release with good biocompatibility and stability. With over 2 years of work by more than 150 experts, a series of TFOS DEWS II reports were published in 2017. This series of reports covered the definition and classification of DE (4), epidemiological reports (1), pathophysiological reports (8), and management and treatment reports (16). Anti-inflammatory drugs were listed as a treatment for DE by the Management and Treatment Report (16). We also noted that the number of articles related to DE anti-inflammatory research had continued to grow since 2017, doubling the number of publications by 2023. Among the top ten co-cited authors Dana Reza, Benitez-Del-Castillo Jose M., Geerling Gerd, and Downie Laura E. co-authored JONES L, 2017, OCUL SURF (16). “JONES L, 2017, OCUL SURF” is located in the top three in the field of DE anti-inflammation both in the LCS and in the GCS index, reflecting its importance in the field and providing important guidance to the field. “*Investigative Ophthalmology & Visual Science*,” “*Ocular Surface*,” and “*Experimental Eye Research*” were the top three in terms of both the number of articles published and the frequency of citations, illustrating their popularity in the field of anti-inflammatory DE and highlighting the extent of its contribution to the field. These journals represented high-quality journals in the field of DE anti-inflammation.

### 4.2 The hotspots and frontiers

We got the research hotspots and frontiers in the field of DE anti-inflammatory research based on keyword co-occurrence, cluster analysis, and trend topic analysis.

#### 4.2.1 Ocular diseases closely related to DE

Since the 1990s, attention was drawn to DE, to the first definition of DE as a disease in TFOS DEWS in 2007 (36), and to the further revision of the definition of DE in TFOS DEWS II in 2017 (4), many progresses had been made in the field of DE, which was closely related to our increasing understanding of the pathophysiology of the disease. The pathophysiologic mechanisms are closely related to the current clinical staging and can also guide clinical treatment.

DE is a complex disease affected by multiple factors that ultimately lead to loss of tear film homeostasis. Loss of tear film homeostasis is the core pathophysiologic basis of DE (8). The ocular surface consists of the cornea, conjunctiva, eyelids, tear film, lacrimal glands, meibomian glands, and so on. The tear film, as part of the ocular surface, is susceptible to interaction with other ocular surface structures. The structure of the ocular surface is a unit, and abnormalities in any of the links associated with tear film stabilization can lead to DE. As a result, diseases that affect the function of the ocular surface are more susceptible to DE.

According to this paper's analysis of the hotspots and trends of keywords, the attention in recent years was mainly on cataract, glaucoma, macular degeneration and other diseases. Stang et al. (37) found that women with eye diseases such as cataract, glaucoma, and macular degeneration were more likely to suffer from DE than men. The prevalence of DE can be as high as 31–36% in patients with allergic conjunctivitis (AC) (38). This could be related to these diseases requiring prolonged use of preservative-containing eye drops. Patients with glaucoma are often treated with intraocular pressure-lowering eye drops, and many of them need a combination of multiple medications. Srivastava et al. (39) has investigated that patients with primary open-angle glaucoma (POAG) who have received medication are more susceptible to the effects of DE. Sedlak et al. (40) also found that in glaucoma patients, the use of benzalkonium chloride (BAC) preserved eye drops increased oxidative stress in the tear film, which may lead to the development of DE. A randomized controlled trial (RCT) was carried out in post-cataract patients, patients with preservative-free dexamethasone eye drops caused less DE symptoms than patients with preservative-containing eye drops (41). Eye drops are a popular method of administration in ophthalmology and are commonly used in many eye diseases and ophthalmic surgeries. Preservatives are often used in eye drops to help preserve them, the most common being BAC. Riedlová et al. (42) demonstrated that BAC impairs the stability of tear film models by constructing *in vitro* and computer models. TFOS DEWS II in a medical report (43) also noted that topical medications themselves can cause DE. Preservatives in medications, such as BAC, may further exacerbate DE. In addition, BAC is now commonly used to induce the formation of DE in animal models, promoting corneal and conjunctival pro-inflammatory cytokine expression, epithelial cell apoptosis, and mucin reduction (44), which can result in mitochondrial damage (14), apoptosis, oxidative stress (45), and pyroptosis (10). Currently, antioxidants, such as glutathione (GSH) (46), and herbal extracts, such as Astragalus extract (47) and artemisinin analogs (48), have been studied to counteract ocular surface damage caused by BAC. The damage to the ocular surface caused by BAC is receiving increasing attention and preservative-free eye drops are being developed. The need for preservative-free medications is even more evident for diseases that require long-term application of eye drops, such as DE and glaucoma.

In addition, eye diseases closely related to DE have shown similarities in pathogenesis. DE, cataract, glaucoma, AMD and diabetic retinopathy (DR) are all age-related eye diseases and their molecular mechanisms are closely related to oxidative stress and inflammation (49, 50). Increased levels of ROS and the oxidative damage they cause play an important role in the aging process (49). The anatomy of the eye, both anterior and posterior segments, are extremely susceptible to ROS. When ROS are overexpressed, oxidative stress occurs, leading to cellular damage, chronic inflammation and tissue degeneration (51). In patients with DE, tissue damage due to oxidative stress can exacerbate inflammation and cellular stress responses, which can further increase ROS levels. Therefore, ROS plays a central role in the vicious cycle of DE inflammation, and antioxidants are now a potential target for the treatment of DE (52). Therefore, targeting antioxidants show promising applications in many ophthalmic diseases.

#### 4.2.2 Mechanisms of inflammation in DE

The complex pathogenesis of inflammation in DE is not fully known. Anti-inflammatory mechanisms in DE have been of interest to researchers. Based on keyword clustering and keyword emergence analysis, it was found that in recent years, researchers have focused on the mechanism of NLRP3 inflammasome and oxidative stress in DE. The NLRP3 Inflammasome is an innate immune system protein complex that plays an important role in DE. Increased expression of NLRP3 inflammasome and its downstream inflammatory factors caspase-1, IL-1β and IL-18 were detected in the tear fluid of DE patients (9). Pyroptosis is a necrotic and inflammatory programmed cell death that plays an important role in the immune response (53). Activation of NLRP3 inflammasome triggers activation of caspase-1 to promote the proteolytic cleavage of gasdermin D (GSDMD) to execute pyroptosis, which is a key link in the classical pathway of pyroptosis (54). GSDMD-dependent pyroptosis has been shown to play a critical role in mouse models of DE (55). Increased pyroptosis was also detected in the tear fluid of DE patients (56).

Targeting NLRP3 inflammatory may provide a new strategy for the treatment of DE. Polydatin, CMC+α-MSH combination therapy, bone marrow mesenchymal stem cell (BMSC), and SM934 have been shown *in vivo* and *in vitro* to attenuate ocular surface inflammatory responses by inhibiting the NLRP3 inflammatory pathway (18, 19, 48, 57). Osteotriol alleviates corneal epithelial cell damage by inhibiting the NLRP3-ASC-caspase-1-GSDMD pyroptosis pathway (56). Treatment with topical dexamethasone effectively inhibits hypertonicity-induced corneal epithelial cell death, possibly through the KCNQ1OT1/miR-214/caspase-1 signal transduction axis (58). In addition, some herbal ingredients, such as Irigenin (59), may also be effective in alleviating cellular pyroptosis in DE models. Electroacupuncture is effective in relieving ocular surface hyperalgesia in patients with DE, possibly by inhibiting the expression of P2X7R and NLRP3-related proteins in the trigeminal ganglion (60).

ROS is located upstream of inflammation. The overexpression of ROS, with an imbalance in the antioxidant system can lead to oxidative stress. Oxidative stress plays an important role in the pathogenesis of DE and may damage the ocular surface by disrupting lipid peroxidation of membranes, oxidative modification of proteins, and causing oxidative damage to DNA (61). In a meta-analysis (62) that included nine articles, patients with DE had higher oxidative stress biomarkers detected in tears and conjunctiva than healthy controls. The (superoxide dismutase) SOD enzyme family is an important antioxidant system. Cu, Zn-superoxide dismutase 1-deficient mice [Sod1(–/–) mice] can lead to an increase in DNA oxidative stress biomarkers in the conjunctiva, as well as a decrease in the expression of goblet cell, mucin expression (63). Nuclear factor erythroid derived-2erelated factor 2 (Nrf2) can regulate the expression of antioxidant and detoxification genes and initiate antioxidant and repair processes. An increase in biomarkers of inflammation and oxidative stress, as well as follicular cell apoptosis, was observed in lacrimal gland tissues of Nrf2 deficient (Nrf2–/–) mice (64). The ocular surface is directly related to the external environment and is susceptible to external environmental influences. Pollution, whether outdoor environmental pollution such as ozone, NO2, and particulate matter, or indoor environment-related pollution, may cause oxidative stress and inflammation production on the ocular surface, triggering DE (65). As induced by the external environment, the production of ROS can trigger the activation of NLRP3 inflammatory and the increased secretion of IL-1 β, which triggers DE (11).

There is a lot of research focusing on targeting antioxidants in an attempt to manage DE from upstream of inflammation. Lactoferrin (Lf), a protein found in tear fluid, is a potential biomarker in the diagnosis of DE (66). LF has anti-inflammatory and antioxidant effects and has been shown to improve the functioning of tissues such as the corneal epithelium and the lacrimal gland (67, 68). In addition, some natural antioxidants have shown promising applications in DE. Quercetin and resveratrol exhibit anti-inflammatory and antioxidant effects on human conjunctival and corneal epithelial cells and may be therapeutic targets for inflammatory ocular surface diseases (69). Quercetin, as a type of polyphenol, has shown excellent antioxidant properties. Quercetin could increase the levels of SOD-1 and SOD-2 in mouse lacrimal gland tissues and increase tear secretion, and could also improve the stability of the tear film in healthy subjects (70). Resveratrol could alleviate ocular surface damage in DE mice by promoting the expression of Mammalian sirtuin 1 (SIRT1) and restoring mitochondrial function (71). Curcumin could effectively inhibit inflammation through multiple signaling pathways (72). The translation of curcumin into the clinic remains difficult with low bioavailability. There has been some research into loading the drug through drug delivery systems such as nano-emulsion to improve the efficacy (73).

#### 4.2.3 Anti-inflammatory treatment in DE

##### 4.2.3.1 Anti-inflammatory drugs

The anti-inflammatory treatment of DE is currently being emphasized by an increasing number of doctors. Attempts have been made to break the vicious cycle of DE with anti-inflammatory drugs, the main anti-inflammatory drugs include non-steroidal anti-inflammatory drugs (NSAIDs), glucocorticosteroids, and immunosuppressive drugs. NSAIDs are commonly used in ophthalmology to reduce ocular inflammation and pain after surgery (74, 75), alleviate ocular signs and symptoms in patients with DE, and reduce ocular surface inflammatory markers (76). However, topical application of pranoprofen eye drops has been reported to delay corneal epithelial cell healing (77) and promote corneal stromal cell apoptosis (78), so care should be taken to avoid application in patients with epithelial damage. Topical glucocorticosteroids have been used to treat a variety of eye diseases, including DE. While exerting anti-inflammatory and immunosuppressive effects (79), glucocorticoids may also relieve nerve pain (80). Fluorometholone eye drops are a class of low-concentration glucocorticosteroids that are effective in relieving symptoms and signs in patients with DE (81). In addition to its anti-inflammatory effects, flumethasone eye drops may also treat DE by increasing the expression of mucins in conjunctival and corneal epithelial cells (82). However, topical use of flomilon eye drops may bring about corneal epithelial cell damage, which may be induced by the preservative (BAC) contained within them (83). In addition, prolonged use of glucocorticoids may increase trabecular meshwork outflow resistance leading to elevated intraocular pressure secondary to glaucoma (84), and may also induce posterior subcapsular cataracts (PSCs) (85). Cyclosporin A, an immunomodulator, is the first FDA-approved anti-inflammatory treatment for DE (86), inhibiting T-cell activation and cytokine production (87), suppressing apoptosis of conjunctival epithelial cells, preventing cupping cell loss (88), and promoting tear secretion (89). For patients with DE after refractive surgery, topical 0.05% cyclosporine A(CsA) combined with 0.1% sodium hyaluronate was effective in reducing ocular surface inflammation and relieving ocular pain (90). However, the use of CsA in clinical settings may result in eye irritation complications such as redness, burning sensation, and itching in the eye (91).

##### 4.2.3.2 Ocular drug delivery systems

Traditional ophthalmic drug formulations suffer from low bioavailability in addition to some side effects of the drug itself (92). In addition, factors such as tear flushing and the corneal barrier also make the bioavailability of ocular drugs < 5 percent (93). Whereas, DE, as a chronic eye disease, requires long-term application of medications, the prolonged use of these medications can cause many side effects, so the long-term treatment of DE remains a challenge. To overcome this challenge, ODDS is attempting to overcome the limitations associated with traditional formulations. ODDS can deliver drugs to target tissues through the ocular barrier to improve therapeutic precision (94); prolong the retention time of drugs on the ocular surface to improve drug bioavailability; reduce the amount of drug administered to mitigate drug side effects (95); and enable multiple drug combinations (96). Popular drug delivery carrier structures used in DE anti-inflammatory therapy include nanoscale (89), liposomes (96), and contact lenses (92). Some drugs with anti-inflammatory and antioxidant properties carry drug delivery systems that could improve drug stability and bioavailability, for example, LF can be loaded in contact lenses and biodegradable polymer nanocapsules (97), as well as in liposomes (98).

##### 4.2.3.3 Physiotherapy

Physical therapy is also a hot topic in DE treatment, and according to keyword clustering analysis and combined with trend theme analysis, during the last 2 years people have begun to pay attention closely to the anti-inflammatory effects of pulsed light therapy (IPL) in DE. DE can be classified as aqueous-deficient dry eye (ADDE) and evaporative dry eye (EDE), with hyper-evaporative dry eye being more common in clinical practice, and meibomian gland dysfunction (MGD) being the main cause of EDE (8). MGD is a multifactorial disease with lid inflammation, microbial growth, associated skin conditions and potentially serious corneal complications, all of which can lead to blockage, detachment or inflammation of the lid glands, ultimately leading to a vicious cycle of DE (99). Current treatments for MGD include topical heat packs, blepharoplasty massage, topical medications, and topical physiotherapy such as IPL and thermal pulsation treatments. IPL is a broad-spectrum pulsed light that was first used in dermatology for its selective photothermal effect that seals off blood vessels and is often used to treat dilated facial capillaries and erythema caused by rosacea (100). The application of IPL in ophthalmology began in 2002 when Professor Toyos found that patients' symptoms of MGD and DE were also relieved during the application of IPL for the treatment of rosacea. MGD is the disease for which IPL is most commonly used in ophthalmology currently. IPL treatment is also listed by TFOS DEWS II as a treatment for DE (16). IPL has demonstrated good efficacy in the treatment of MGD, and its mechanism of action may be related to the fact that IPL improves meibomian gland function and attenuates the expression of inflammatory factors in the tear fluid (101), the heat generated by IPL melts meibomian gland blepharoplasty and dilates the glands (102), reduces helminth mites at the margins of the lids (103, 104), and restores the hypoxic environment of meibomian glands (105). The light energy released by IPL can be absorbed by melanin, hemoglobin and water, which in turn is converted into heat (106). Eye tissues such as the iris are rich in pigment and are susceptible to absorbing light in the IPL wavelength range and being affected. Serious ocular complications such as iritis and pupil abnormalities have been reported with IPL treatment of the face (107). Hence, attention should be paid to eye protection during the use of IPL. In addition, the higher economic cost is also a challenge for IPL clinical applications (108).

### 4.3 Limitations

This study is based on the bibliometric approach to explore the field of DE anti-inflammatory studies, the number of publications, countries, institutions, authors, journals, references, and keywords in the field are visualized to facilitate the researchers to quickly understand the research hotspots and frontiers in the field. However, this study also has some limitations. Firstly the analysis only included English-language articles, and the impact of other non-English-language articles may have been underestimated. Secondly, we restricted the publication of articles to those published in the last 20 years, which may have resulted in an inability to fully cover all the hot topics in the field of DE anti-inflammatory studies. In addition, the database searched was WOSCC, and articles from other database sources have not yet been included in the study. It is hoped that in the near future, the output formats of different databases can be standardized so that the databases can be better merged, which is conducive to summarizing and analyzing, and a better understanding of the research profile in this field. Finally, the bibliometric analysis software was unable to analyze the specific content of the included articles. Of course, these issues may be some of the problems inherent in the bibliometric research methodology. Therefore, we had read and compiled a large amount of paper in order to comprehensively analyze the research trends and hotspots in the field of DE anti-inflammatory studies, so that the researcher could have a quick overview of the field.

## 5 Conclusion

Inflammatory mechanisms and anti-inflammatory treatment in DE are the hotspots in this field. This study provides the first bibliometric analysis of trends in DE anti-inflammatory studies over the past two decades. Overall, there has been a steady increase in the number of publications in the field of DE anti-inflammatory studies, and active collaborations are maintained worldwide. Targeted therapy of inflammation, application of drug delivery systems, and physical therapy such as ILP may be future research directions. Our study systematically summarizes and analyses the research hotspots and emerging trends in the field, which can enable clinicians and researchers to better grasp the direction of research.

## Data Availability

The original contributions presented in the study are included in the article/Supplementary material, further inquiries can be directed to the corresponding authors.
