# Supplementary material for: Global research hotspots and trends in anti-inflammatory studies in dry eye: a bibliometric analysis (2004–2024)
Source: Front Med (Lausanne). 2024 Nov 28;11:1451990. doi: 10.3389/fmed.2024.1451990 (PMC11634594; doi:10.3389/fmed.2024.1451990)
Supplement: Supplementary file 1 [file Data_Sheet_1.pdf]

Web of Science Search Strategy (v0.1)

Database: Web of Science Core Collection

Entitlements:

- WOS.IC: 1993 to 2024
- WOS.CCR: 1985 to 2024
- WOS.SCI: 2003 to 2024

Searches:

1: TS=(anti inflammatory* OR anti-inflammatory* OR anti-inflammation*) Date Run: Sun May 05 2024 09:45:32 GMT+0800 (N-Výh QÆeö•ô) Results: 266275

2: TS=(Dry Eye* OR Dry Eye Syndrome* OR Dry Eye Disease* OR Evaporative Dry Eye*) Date Run: Sun May 05 2024 09:45:53 GMT+0800 (

3: #2 AND #1 Date Run: Sun May 05 2024 09:46:03 GMT

4: #2 AND #1 Timespan: 2004-01-01 to 2024-05-05 Date Run: Sun May 05 2024 09:46:35 GMT+0800 (N-Výh QÆeö•ô) Results: 654

5: #2 AND #1 and English (Languages) Timespan: 2004-01-01 to 2024-05-05 Date Run: Sun May 05 2024 09:47:10 GMT+0800 (N-Výh QÆeö•ô) Results: 627

6: #2 AND #1 and English (Languages) and Article or Review Article (Document Types) Timespan: 2004-01-01 to 2024-05-05 Date Run: Sun May 05 2024 09:47:16 GMT+0800 (N-Výh QÆeö•ô) Results: 603
