# Supplementary material for: Global research hotspots and trends in anti-inflammatory studies in dry eye: a bibliometric analysis (2004–2024)
Source: Front Med (Lausanne). 2024 Nov 28;11:1451990. doi: 10.3389/fmed.2024.1451990 (PMC11634594; doi:10.3389/fmed.2024.1451990)
Supplement: Supplementary file 2 [file Table_1.DOCX]

The proposed items of PRIBA*

| **Section and topic** | **Item**  **No** | **Proposed item to be used in bibliometric research** | **Page**  **No** | **Line numbers in the manuscript** |
| --- | --- | --- | --- | --- |
| **Title** |  |  |  |  |
| Title | 1a | Identify the study is a bibliometric analysis. | 1 | 1 |
|  | 1b | Indicate the coverage period. | 1 | 1 |
| **Abstract** |  |  |  |  |
| Abstract | 2a | Provide an explicit statement of objective(s). | 1 | 14-16 |
|  | 2b | Specify the data sources. | 1 | 17 |
|  | 2c | Specify the coverage period. | 1 | 18 |
|  | 2d | Provide results for main outcomes. | 1 | 20-29 |
|  | 2e | Provide an overall interpretation of the results and implications. | 1 | 30-32 |
| **Introduction** |  |  |  |  |
| Rationale | 3 | Describe the rationale for the study. | 2 | 34-63 |
| Objectives | 4 | Provide an explicit statement of objective(s). | 2 | 64-71 |
| **Methods** |  |  |  |  |
| Data source | 5a | Specific the database(s) or other data sources searched. | 2 | 74 |
|  | 5b | Describe the characteristics of the data source. | 2 | 74-76 |
|  | 5c | Specify the date when the search was conducted. | 3 | 78-79 |
| Eligibility criteria | 6 | Specify the inclusion and exclusion criteria, such as language, article types, and coverage period. | 3 | 79-80 |
| Search strategy | 7 | Specify the search strategy and keywords used. | 2-3 | 76-79 |
| Bibliometric indicators | 8 | Describe the bibliometric indicators used. | 3 | 90-115 |
| Analytical software | 9 | Specify the software package(s) used and the settings selected. | 3 | 85-90 |
| **Results** |  |  |  |  |
| Search process | 10 | Describe the results of the search and selection processes, and use a flow diagram if necessary. | 3 | 82-83 |
| Bibliometric indicators | 11 | Describe the results of bibliometric indicators, including quantity, performance, and structural indicators. | 3-7 | 116-259 |
| Figures | 12 | Prepare figures with an adequate resolution for online and print readability. | 18-21 | 804-816 |
| **Discussion** |  |  |  |  |
| Conclusions | 13 | Summarize key results with reference to study objective(s). | 7-8 | 261-299 |
| Limitations | 14 | Discuss any limitations and impact of potential bias. | 11-12 | 471-487 |
| Interpretation | 15 | Interpret the results in the context of background knowledge. | 8-11 | 300-470 |
| **Other information** |  |  |  |  |
| Support | 16 | Describe the sources of financial or non-financial support and the role of funders or sponsors. | 12 | 500-503 |
| Conflicts of interest | 17 | Declare any competing interests of the author(s). | 12 | 497-499 |
| Data availability | 18 | Specify if data are publicly available and the route of access. | 12 | 508-510 |

*PRIBA: Preferred Reporting Items for Bibliometric Analysis.

PRIBA is the first PRISMA Checklist of bibliometric studies. This table is based on the paper by Koo M and Lin SC. (<https://doi.org/10.1016/j.heliyon.2023.e16780>).
